# Behavioral Modification of *Leucauge mariana* Induced by an Ichneumonid Spider-Parasitoid, *Hymenoepimecis castilloi*, in the Colombian Andes

**DOI:** 10.1007/s13744-023-01110-9

**Published:** 2023-12-22

**Authors:** Andrés F. Velasco-Cárdenas, Jesús C. Jacome-García, Diego G. Pádua, Thiago G. Kloss

**Affiliations:** 1 Grupo Diversitas, Facultad de Ciencias Básicas y Aplicadas, Univ Militar Nueva Granada, Cajicá, Colombia; 2 Grupo Exploratorium, Fundación Clínica Shaio, Bogotá, D.C. Colombia; 3 Programa de Pós-Graduação Em Entomologia, Instituto Nacional de Pesquisas da Amazônia – INPA, Manaus, Amazonas Brazil; 4 Centro de Investigación de Estudios Avanzados del Maule, Vicerrectoría de Investigación y Postgrado, Univ Católica del Maule, Talca, Maule, Chile; 5 Lab of Behavioral Ecology, Dept of General Biology, Federal University of Viçosa, Viçosa, Minas Gerais Brazil

**Keywords:** Andean Forest, Cocoon web, Darwin wasps, Ectoparasitoid, Koinobiont, Tetragnathidae

## Abstract

*Hymenoepimecis* is a genus of Darwin wasps in the *Polysphincta* group of genera (Hymenoptera: Ichneumonidae: Pimplinae) known as ectoparasitoids of a broad spectrum of spiders. The parasitoid induces production of a web known as cocoon web, which provides shelter and support for the wasp pupa. In this study, we describe for the first time the interaction between *Hymenoepimecis castilloi* Pádua & Sääksjärvi (Hymenoptera: Ichneumonidae) and its host spider *Leucauge mariana* (Taczanowski) (Araneae: Tetragnathidae) in the Colombian Andes, provide new records of wasp genus distribution, and described the behavioral modifications induced in the spider. Web modifications occurred in the webs of both solitary and aggregated individuals. Adhesive spirals were lacking, and webs were connected to vegetation by multiple threads in all cocoon webs, which was not seen attached to webs of non-parasitized spiders. All parasitoid cocoons were observed hanging on a vertical line in the hub of the cocoon web. As previously described for other species, we believe that this modified web design results in increased web strength and favors parasitoid development during the pupal stage.

## Introduction

Some parasitoids have developed the ability to induce behavioral changes in their hosts, which increases the probability of surviving during their relatively vulnerable pupal stage (Grosman et al. 2008; Gonzaga et al. 2017; Kloss et al. 2018). Ichneumonidae or Darwin wasps (Klopfstein et al. 2019) of the *Polysphincta* genus group (sensu Gauld and Dubois 2006), hereafter polysphinctines, represent an example of parasitoids that induce behavioral modifications in their host spiders. These wasps are koinobiont ectoparasitoids of nine spider families, mainly juveniles or sometimes adults (Eberhard and Gonzaga 2019; Gaione-Costa et al. 2022). Behavioral modifications are characterized by a modified web design to support the wasp larvae cocoon (cocoon web), which serves as a shelter for the wasp pupae (Eberhard 2001; Korenko et al. 2015; 2022). This modified behavior can be triggered in some interactions by enforced activation of ecdysis mode, which is characterized by construction of modified webs that normally act as protection for the spiders (Takasuka et al. 2015; Kloss et al. 2017; Eberhard and Gonzaga 2019).

Polysphinctine wasps include 25 genera, and some genera are exclusive to the Neotropical region, like *Hymenoepimecis* Viereck. This genus includes 28 species, which are distributed in low and mid altitudes from tropical Mexico to northern Argentina (Yu et al. 2016; Pádua et al. 2020; Pádua 2022). Information about host behavior modification was described to 12 species, associated with spiders of the Araneidae and Tetragnathidae families (Eberhard and Gonzaga 2019; Gaione-Costa et al. 2022; Kloss et al. 2022; Santos-Murgas et al. 2022), especially of the genus *Leucauge* White (Araneae: Tetragnathidae) (Eberhard 2000a, b; Sobczak et al. 2009; Eberhard 2013; Gonzaga et al. 2015; Pádua et al. 2016; Gaione-Costa et al. 2022; Kloss et al. 2022; Santos-Murgas et al. 2022).

Some species of *Hymenoepimecis* were observed using two host species, such as *H. heidyae* Gauld, *H. japi* Sobczak et al., *H. bicolor* (Brullé), and *H. veranii* Loffredo & Penteado-Dias (Gonzaga and Sobczak 2007; Sobczak et al. 2014; Barrantes et al. 2018; Eberhard and Gonzaga 2019; Gonzaga et al. 2022) as well as two wasp species using the same host, such as *H. cameroni* Townes and *H. pinheirensis* Penteado-Dias & Pádua parasitizing *L. volupis* (Keyserling) (Gaione-Costa et al. 2022; Kloss et al. 2022). Cocoon web induced by these species showed variations in their web design, which have been associated with host traits. In addition, variations in web design were described among parasitized individuals of host species *L. volupis* (Keyserling), parasitized by *H. pinheirensis* Townes wasp (Gonzaga et al. 2015; Kloss et al. 2022). The key to understanding the mechanism of manipulation can be associated with the increase of knowledge about interactions of wasps with multiple hosts.

In this study, we described a new case of behavioral manipulation of *Leucauge mariana* and the first case of spider-parasitoid interaction at high altitude (2583 masl) in the Colombian Andes. Previously, *L. mariana* was registered parasitizing *H. tedfordi* Gauld and *Eruga* ca. *gutfreundi* Gauld (Eberhard 2013), and here, we describe a third parasitoid associated with this host spider: *Hymenoepimecis castilloi* Pádua & Sääksjärvi. Additionally, we describe the male of *H. castilloi* that was previously unknown and extend its distribution records for both genus and species.

## Materials and methods

### Study area

**Fig. 1:**
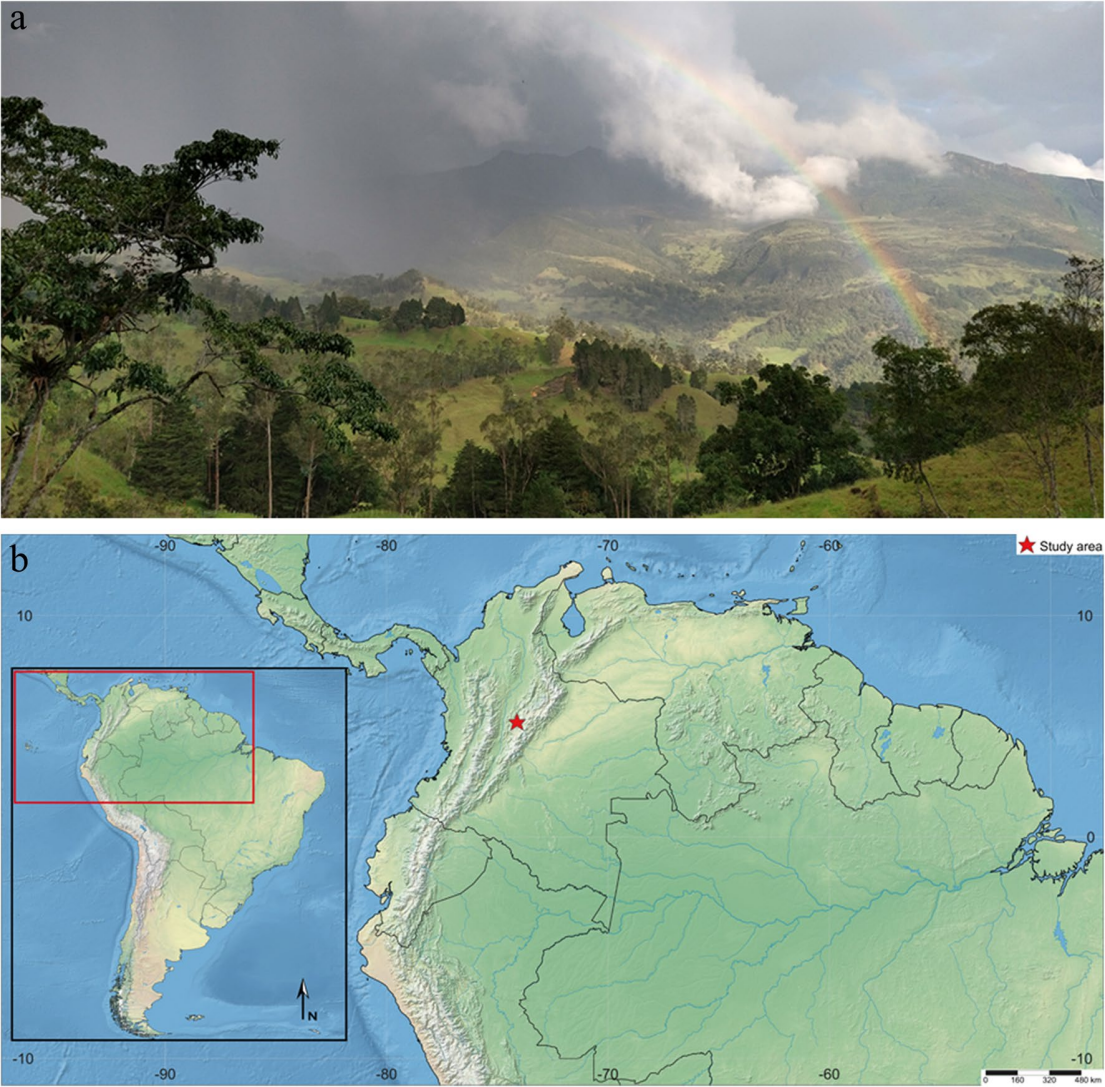
**a** Study area, Pacho, Cundinamarca, Colombia. **b** Map position of the study area

Field observations were made in Pacho, Cundinamarca, Colombia (5°12′13.7″N, 74°06′32.0″W; 2583 m ASL) in March of 2022 (Fig. 1a, b). The study area is mainly used for livestock and small areas for agriculture. The landscape is heterogeneous, with Andean Forest components in different successional stages, immersed in a matrix of grasslands and eucalyptus plantations.

### Host spider species

*Leucauge mariana* has been previously recorded in Mexico and Hispaniola to Peru (World Spider Catalog, 2022). This spider builds more or less horizontal, two-dimensional orb webs. In the study area, individuals occur in aggregates with several webs. Voucher specimens of *L. mariana* were deposited in the arachnid collection at Centro de Coleções Taxonômicas da Universidade Federal de Minas Gerais (UFMG), Belo Horizonte, Minas Gerais, Brazil (A.J. Santos, curator).

### Taxonomy

The parasitoid wasps studied were deposited in the Invertebrate Collection at Instituto Nacional de Pesquisas da Amazônia (INPA), Manaus, Amazonas, Brazil (M.L. Oliveira, curator) and Florida State Collection Arthropods (FSCA), Gainesville, FL, USA (Elijah Talamas, curator). Digital images of wasps were taken using a Leica DMC4500 digital camera attached to a Leica M205A stereomicroscope and multiple layers were stacked by using the software Leica Application Suite V4.10.0. The map was made using SimpleMappr (Shorthouse 2010).

### Behavioral modifications

We present records of the web structure constructed by non-parasitized and parasitized *Leucauge mariana* spiders in the field. We took digital images of the webs using a Canon EOS 80D with a Canon EF35mm lens attached. We analyzed seven webs built by parasitized females and one web of a parasitized male, both parasitized by second instar wasp larvae. We also analyzed seven cocoon webs and three typical prey capture webs built by non-parasitized female spiders. All webs were found on March 24, 2022. To get contrast in the photos, we coated the webs with cornstarch (Eberhard 1976). In all webs, we evaluated the number of radii, spirals, presence of tangle threads below the hub, presence of circular hub lines, and the open hole in the web hub. After taking the photos, we collected parasitized individuals of *L. mariana* and *H. castilloi* pupae. All individuals were kept in plastic pots in a laboratory under controlled conditions (15 ± 2°C and 70 ± 10% HR), located in the Fundación Clínica Shaio Exploratorium Laboratory, until the emergence of adult wasp parasitoids. Finally, we collected five non-parasitized female spiders for species identification.

## Results

### Taxonomy

**Fig. 2:**
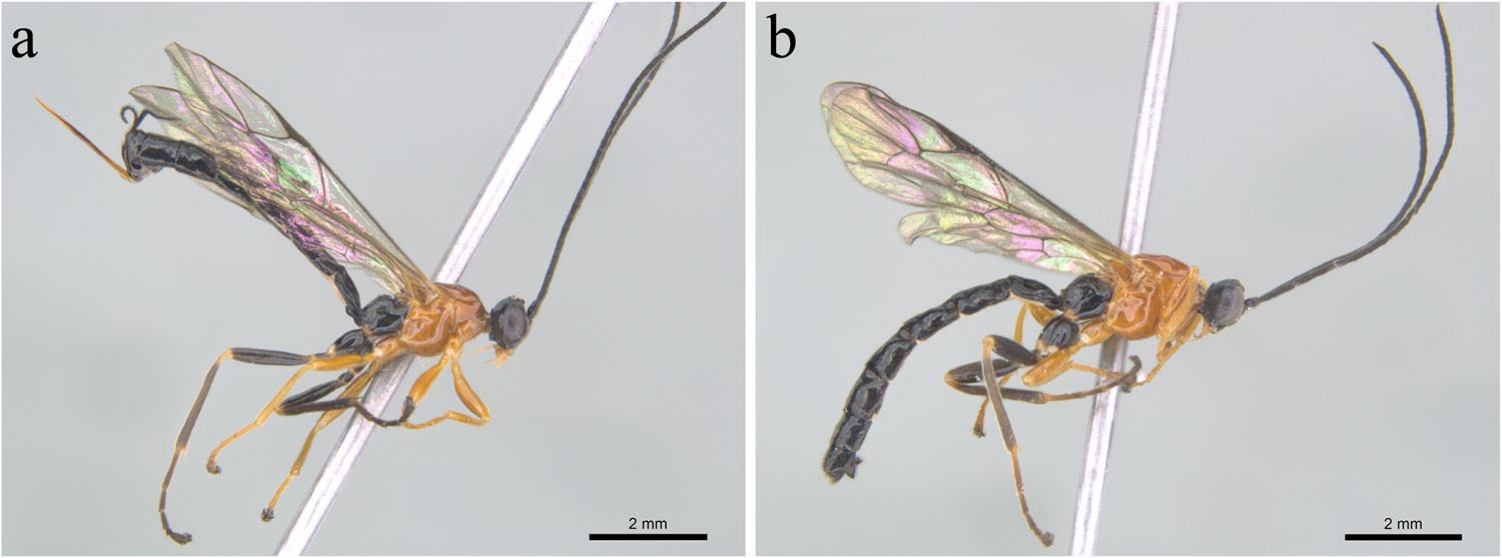
*Hymenoepimecis castilloi* Pádua and Sääksjärvi, 2020: **a** Habitus (♀); **b** Habitus (♂)

*Hymenoepimecis castilloi* Pádua & Sääksjärvi, 2020 (Fig. 2a, b).

**Diagnosis.** See Pádua et al. (2020).

**Male.** (Fig. 2b). Similar to female in structure and coloration, with body 9.0–10.6 mm; face approx. 0.6 times as width as high; posterior ocelli separated from eyes by 0.7–0.8 times its own maximum diameter; fore wing 6.3–7.7 mm; tarsal claw simple; metasoma slender, tergite I 1.4–1.7 times as long as posteriorly width; tergite II 1.25–1.5 times as long as posteriorly width; hypopygium with posterior margin weakly concave.

**Material examined.** COLOMBIA, Cundinamarca, Pacho (5.203806N, − 74.108889W) [= 5°12′13.7″N, 74°06′32.0″W], 2583 mASL, collection of parasitized *Leucauge* sp. [= *L*. *mariana*], 23.III.2022 (A. Velasco and J. Jacome leg.), 6♂♂ and 6♀♀, INPA; Valle [= Valle del Cauca], Arriba Villa Carmelo, 31.VIII.1975 (L. Stange leg.), 1♀, FSCA.

**Distribution.** Argentina; Colombia (new record); Peru.

### Web modification

**Fig. 3:**
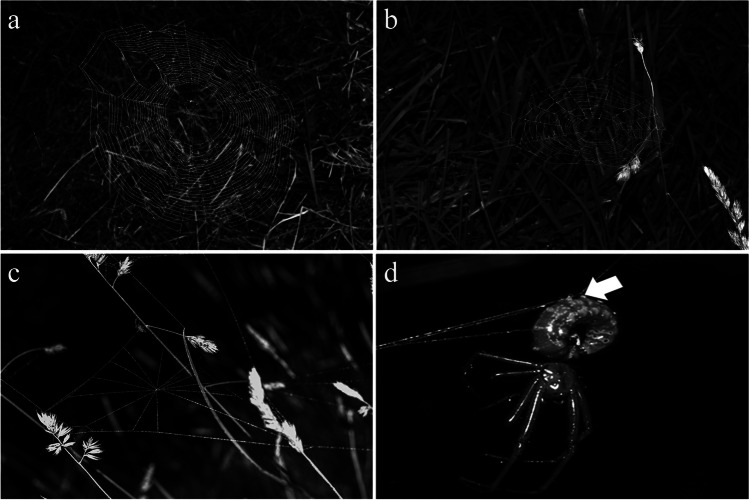
**a** Web of unparasitized individuals of *Leucauge mariana*, absence of tangle threads below the hub. **b** Web of an individual of *Leucauge mariana* parasitized by an *Hymenoepimecis castilloi* second instar larvae. **c** Modified web of parasitized individuals of *Leucauge mariana* by a second instar larvae of *Hymenoepimecis castilloi*, without open hole in the hub and spirals. **d** Adult of *Leucauge mariana* being killed by a third instar larvae of *Hymenoepimecis castilloi*, dorsal tubercles can be seen in the larvae

Typical prey capture webs of non-parasitized spiders had 21.3 ± 9.8 (mean ± S.D.) spirals and 23.6 ± 4.04 radii (*N* = 3, Fig. 3a). Webs of parasitized spiders by second instar wasp larvae had 17.2 ± 8.9 (mean ± S.D.) spirals and 18.5 ± 3.4 radii (*N* = 7, Fig. 3b). All webs of non-parasitized and parasitized spiders by second instar wasp larvae did not present tangle threads below the hub (*N* = 11) (Fig. 3a, c). Also, all webs of non-parasitized spiders and seven webs of parasitized spiders by second instar wasp larvae presented radial lines that converged to a hub with an open hole in the center (Fig. 3a, b). Only one web of parasitized spiders by second instar wasp larvae did not present an open hole in the hub and spirals; however, this web had radial lines that converged to a hub (Fig. 3c). Also, we observed that non-parasitized and parasitized spiders by second instar wasp larvae presented 1–3 circular hub lines (Fig. 3a–c).

**Fig. 4:**
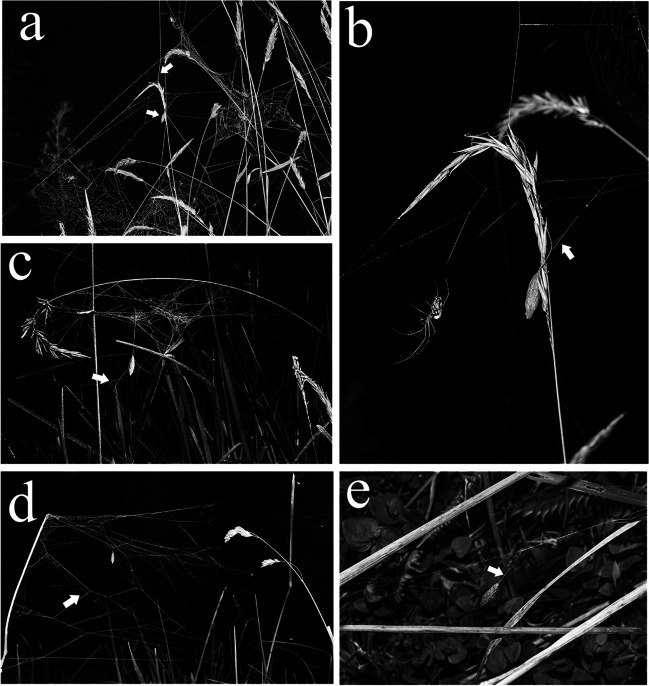
**a **Cocoon web built in aggregates of spiders with the cocoon hanging on the line that connects to all the surrounding webs, with the white arrows indicating the cocoon and the vertical shared frame line which the cocoon is hanging on. **b** An angle-adjusted close-up of the cocoon showed in **a**, exhibiting a clear view of the cocoon hanging from the vertical shared frame line mentioned earlier and the plant flower, with the white arrow indicating the attachment line that is connecting the orange vertical line spun by the larvae and the multiple shared frame lines of the aggregate. **c** A cocoon with a pupa in hanging on a vertical line from a modified cocoon web with a sparse tangle below (white arrow). **d** A cocoon with a pupa in hanging on a vertical line from a modified dense web of *Leucauge mariana*, also a sparse tangle below can be seen (white arrow). **e** Cocoon hanging on a vertical line (white arrow) in a horizontal 2D modified web composed by a few reinforced radii. All cocoon webs had lines connected to vegetation at multiple points to give support to the structure

We observed that all cocoon webs (*N* = 7) of parasitized spiders lacked adhesive spirals, an open hole in the center and hub spiral (Fig. 4a–e). Five cocoon webs were built in web aggregates of spiders, with non-parasitized and parasitized individuals. In these aggregations, all parasitoid cocoons were suspended in a vertical line constructed by the 3rd instar larvae. This line is easily distinguishable due to its orange coloration and thicker texture compared to the normal lines of the spider’s web. The wasp would remain hanging at the exact location where the spider died, and it was common for the spider to die on one of the lines that comprised its own web or one of the sharing frame lines (vertical or horizontal) of other spiders within the aggregation (Fig. 4a, b). Nevertheless, it was not possible to determine which was the original web of these individuals in aggregates because the parasitized spiders in the aggregate moved through the sharing lines to other webs from the aggregate constantly. The place of death of the spiders did not have a pattern of modified web (the webs did not present radial lines that converged to a hub) (Fig. 4a, b). However, two of the five parasitoid cocoons in these aggregates were hanging on a vertical line from an irregular and dense web with a sparse tangle below the hub seemingly constructed by the parasitized spiders. These two webs also did not have reinforced radii converging to a hub (Fig. 4c, d). Finally, we observed two parasitized individuals that built their cocoon webs in the vegetation, away from the aggregates of individuals. These cocoon webs had three and four reinforced radii, which converged to a hub and where the parasitoid hung their cocoon in a vertical line (Fig. 4e). All cocoon webs, if present, had lines connected to vegetation at multiple points (Fig. 4c–e), which was not observed in webs of non-parasitized spiders (Fig. 3a).

## Discussion

We recorded for the first time the presence of the *Hymenoepimecis* genus in Colombia. Previously, Pádua et al. (2015; 2020) and Pádua (2022) studied individuals of this genus that were found in several countries of Central and South America, except in Colombia. A female of *H. castilloi* was previously found in a malaise trap in the Peruvian Andes (Pádua et al. 2020), but its hosts were unknown and now have been confirmed as *Leucauge mariana.* Since Colombia is the biological corridor between Central and South America, many species must be present in this territory.

Eberhard (2013) found that cocoon webs of *L. mariana* parasitized by *Eruga* ca*. gutfreundi* differ from those parasitized by *H. tedfordi*. The main differences were that *Eruga* ca*. gutfreundi* cocoon webs showed a 3D structure with multiple radial lines radiating in many directions that converged in the cocoon, while *H. tedfordi* cocoon webs were planar with strengthened radial and frame lines, occasionally presenting a sparse tangle of lines below. We observed that two *H. castilloi* cocoon webs found in the web aggregations (Fig. 4c, d) presented a cocoon hanging on a vertical line with a sparse tangle below. However, unlike those of *H. tedfordi*, the *H. castilloi* cocoon webs exhibited an irregular structure instead of being planar. Also, the sparse tangle below cocoon web type was previously reported in other species like *H. pinheirensis* parasitizing *L. volupis* (Kloss et al. 2022). However, the two simpler cocoon webs found away from the web aggregates for *H. castilloi* (Fig. 4e) were more similar to the typical cocoon web design of *L. argyra* (Walckenaer) parasitized by *H. argyraphaga* Gauld (Eberhard 2001) and to those highly reduced cocoon webs of *H. tedfordi* (Eberhard 2013) with only a few reinforced radii converging to the hub.

The complete lack of adhesive spirals and the increase of lines connected to vegetation in order to reinforce the modified web of *L. mariana* were observed in all interactions between *Leucauge* and *Hymenoepimecis* wasps (Eberhard 2001; Sobczak et al. 2009; Gonzaga et al. 2015; Pádua et al. 2016; Santos-Murgas et al. 2022; Kloss et al. 2022). We suggest that these features of cocoon webs may promote protection against scavenging arthropods, stability against climate conditions, and flying insect strikes, as previously observed in cocoon webs built by *Agelena silvatica* Thorell (Araneae: Agelenidae: formerly *A. limbata*) (Matsumoto 2009), *L. volupis* (Gonzaga et al. 2015), and by spiders of genus *Cyclosa* Menge (Araneae: Araneidae) (Matsumoto and Konishi 2007; Takasuka et al. 2015; Kloss et al. 2016).

We found variation among *H. castilloi* cocoon webs based on the web pattern. When cocoon webs occur in web aggregates, there were shared vertical or horizontal lines depending on the structure of the aggregation (different from the orange strand that the larvae produce to hang from the spider web) that connected the surrounding inmates webs, in which we found the cocoon hanging (Fig. 4a) or an irregular and dense web with a sparse tangle below the hub (Fig. 4c, d). However, the solitary cocoon web was formed by 3 to 4 reinforced radii converging to a hub (Fig. 4e), similar to the cocoon webs made by *L. argyra* (Eberhard 2001), *L. roseosignata* Mello-Leitão (Sobzack et al. 2009), *L. henryi* Mello-Leitão (Pádua et al. 2016), and *L. venusta* (Walckenaer) (Santos-Murgas et al. 2022) under the influence of *Hymenoepimecis* species. In the solitary cocoon webs and in aggregates of *L. mariana* webs, the parasitoid cocoons were always hanging on a vertical line built by itself, and the web had lines connected to the vegetation at multiple lines. *Leucauge* individuals sometimes cooperate and can group under specific conditions forming colonies (Salomon et al. 2010). These colonies consist of individual orb webs connected by a shared framework silk that are anchored to the vegetation with thick silk threads, and we found the cocoons hanging on these structures. We suggested that this structure is stable enough that it does not need reinforcement, resulting in the less evident cocoon web, while in solitary webs there is a clear pattern of a modified web with reinforced silk. However, regardless of the construction site, the observed patterns in cocoon webs suggest that it has a higher resistance than normal, being suitable for the development of parasitoids during the pupal stage.

According to Kloss et al. (2016), one of the most important reasons for decreasing pupae survivorship is rain and any falling object that can break the web structure and make the pupae fall to the ground where they are at higher risk of predation. Also, there is an intraspecific variation in the cocoon web of *Leucauge mariana* that can be found with modification when it is solitary, and without a pattern of modified web or occasionally an irregular web with a sparse tangle below when is in a web aggregate. Following these findings, there is not only a difference in cocoon webs between different *Hymenoepimecis*-*Leucauge* species interactions but also in different population conditions.

We noticed that *H. castilloi* and *H. tedfordi* (Eberhard 2013) manipulated *L. mariana* to build similar cocoon webs when they were solitary. On the other hand, *L. mariana* built a different type of cocoon web under the influence of *E.* ca. *gutfreundi* (Eberhard 2013). However, we also found that different conditions in the spider population (aggregates and solitary) can change the building pattern of the cocoon webs. Further investigations controlling the situations of manipulation (e.g., making parasitized spiders in a web aggregation solitary just before manipulation will occur) are needed to obtain a clue to the switching mechanism of cocoon web pattern.
